# Multidimensional team performance indicators in men's UEFA EURO 2024: an exploratory analysis

**DOI:** 10.3389/fspor.2026.1955357

**Published:** 2026-09-21

**Authors:** Swamynathan Sanjaykumar, Ponnusamy Yoga Lakshmi, Priyanka Rana, Sergio Sebastia-Amat, Zhanneta Kozina, Gowri Anil, Jun Mizushima

**Affiliations:** 1 Department of Bachelor of Physical Education, Christ College (Autonomous), University of Calicut, Irinjalakuda, India; 2 Department of Computer Science, Faculty of Science and Humanities, SRM Institute of Science and Technology, Kattankulathur, India; 3 Department of Physical Therapy and Movement Science, University of Texas at El Paso, El Paso, TX, United States; 4 Department of General and Specific Didactics, Faculty of Education, Health, Physical Activity and Sports Technology (HEALTH-TECH), University of Alicante, Alicante, Spain; 5 Department of Olympic and Professional Sport, Sport Games and Tourism, H.S. Skovoroda Kharkiv National Pedagogical University, Kharkiv, Ukraine; 6 Department of Computer Application, School of Computer Science Engineering and Information Systems, Vellore Institute of Technology, Vellore, India; 7 Department of Health and Sports Sciences, Faculty of Health and Sports Sciences, Toyo University, Tokyo, Japan

**Keywords:** football analytics, machine learning, performance analysis, team performance indicators, UEFA EURO

## Abstract

This study examined multidimensional team-level performance indicators from men's UEFA EURO 2024 and evaluated whether they could support exploratory classification and descriptive benchmarking. Publicly available data from all 24 participating teams were analysed using seven retained indicators representing possession, attacking output, expected performance, and progression. Relationships among indicators were examined, and a composite Team Strength Score (TSS) was calculated from normalised retained indicators after cumulative counts were expressed per 90 min. Exploratory classification accuracy ranged from 0.50 to 0.58 and did not exceed the 0.67 majority-class baseline. The strongest correlations were observed between goals and assists (r = 0.91), xG and xA (r = 0.90), progressive carries and progressive passes (r = 0.89), and possession and progressive passes (r = 0.89). These associations partly reflect intrinsic relationships among football actions and should not be interpreted as independent determinants of performance. Spain and Germany had the highest TSS values. The framework may support post-tournament benchmarking, opponent analysis, and communication between performance analysts and coaching staff, provided that possession, progression, and expected-performance indicators are interpreted alongside realised outcomes and match context.

## 1 Introduction

Association football is a complex team sport in which performance emerges from the interaction of technical actions, tactical organisation, and decision-making under pressure. This complexity is especially evident in short international tournaments, such as the UEFA European Championship (UEFA EURO), where national teams compete across a small number of matches and where small differences in attacking efficiency, ball progression, and defensive organisation can affect tournament outcomes (1, 2). Traditional indicators, such as goals scored, possession rate, and match results, provide useful information, but they do not fully capture how teams create chances, progress the ball, and sustain attacking pressure across a tournament (3).

The rapid development of football analytics has expanded opportunities for the quantitative assessment of team performance (4). For example, expected goals (xG) estimate the probability that shots will result in goals, while expected assists (xA) estimate the expected assist value of passes. In addition, progressive carries and progressive passes provide information about how teams move the ball towards goal and create territorial advantage. These indicators allow analysts to move beyond isolated match outcomes and examine the processes that contribute to team performance (5, 6). However, the interpretation of these indicators remains challenging when they are examined separately, because many technical and tactical variables are interrelated.

Previous key performance indicator (KPI) research indicates that performance indicators in elite football are context dependent rather than universal. Wright et al. (7) showed that goal-scoring outcomes depend on the origin and characteristics of attacking sequences, while Winter and Pfeiffer (8) identified transition play and offensive efficiency as discriminative tactical dimensions in UEFA EURO 2012. Mitrotasios et al. (9) demonstrated that routes to goal-scoring opportunities differ across major European leagues, and Mendes et al. (10) showed that specific restart strategies can be associated with subsequent offensive outcomes. More recently, Martín-Castellanos et al. (11) reported that the relevance of distribution, attacking, defending, goalkeeping, and disciplinary KPIs varied between the group and knockout stages of UEFA EURO 2024. Together, these studies support a multidimensional and context-sensitive interpretation of football KPIs rather than a single deterministic set of success variables.

Machine learning methods provide one possible approach for organising multidimensional performance data (12–14). Logistic Regression, Random Forest, Support Vector Machine (SVM), and Extreme Gradient Boosting (XGBoost) have been used in sports analytics to classify performance patterns and explore relationships among multiple variables (4, 15, 16). These methods can support performance analysis by identifying patterns that may not be apparent when indicators are examined in isolation. However, classification accuracy may be sensitive to the number of observations, the definition of the outcome variable, and the validation procedure. Therefore, machine learning should be used as an exploratory tool rather than as a definitive prediction system when only team-level data from one tournament are available.

International football tournaments provide a useful setting for exploratory team-level performance analysis because participating teams compete within the same competition structure (1). UEFA EURO 2024 included 24 national teams and offered a recent dataset of team-level technical and tactical indicators (17). Analysing these indicators can help identify differences in attacking output, chance creation, ball progression, and possession across teams (18). A transparent analytical framework may also help performance analysts and coaching staff communicate tournament findings and compare team strengths in a structured way. Such an approach is valuable when it is used for post-tournament review, opponent analysis, and benchmarking rather than for the deterministic prediction of future results. Compared with domestic leagues, a single international tournament provides a more bounded competitive setting because all teams are evaluated within the same competition format and a narrow time window, although this advantage is offset by the substantially smaller number of team-level observations.

Composite indicators may be useful for summarising multidimensional team performance. Prior KPI studies have shown the value of considering multiple technical and tactical domains together rather than relying on a single outcome variable (1, 11). In the present study, a Team Strength Score was developed as a transparent descriptive summary of the retained normalised indicators. This type of score does not replace detailed tactical analysis, but it can provide an overview of how teams compare across several performance dimensions. Its practical value lies in directing analysts back to the contributing indicators for more detailed match-specific interpretation rather than in defining a tactical profile by itself.

The present study therefore addresses a methodological gap by integrating exposure-adjusted multidimensional performance indicators, their correlation structure, exploratory classification, and a transparent descriptive composite benchmark within a single tournament-level framework while explicitly accounting for outcome leakage.

Accordingly, this study aimed to examine multidimensional team-level performance indicators from men's UEFA EURO 2024. The specific objectives were to: (i) examine relationships among selected technical and tactical performance indicators; (ii) compare selected machine learning models as supplementary exploratory classification tools; and (iii) calculate a composite Team Strength Score to rank national teams descriptively.

## 2 Methods

### 2.1 Research design

This study used an exploratory, secondary data analysis design. The analysis was based on publicly available team-level performance statistics from UEFA EURO 2024. The purpose of the analysis was to examine multidimensional team-level performance indicators and to determine whether selected machine learning models could provide supplementary classification information. The analysis was conducted at the team level, and each national team represented one observation.

### 2.2 Data source and sample

The dataset consisted of performance statistics for the 24 national teams that participated in UEFA EURO 2024. The data were obtained from publicly available UEFA EURO 2024 team statistics provided by FBref (17). All 24 participating teams were included in the analysis, and no team was excluded.

The unit of analysis was the national team rather than the individual player or match. Therefore, the findings should be interpreted as team-level tournament summaries rather than match-level or player-level estimates.

### 2.3 Performance indicators

The selected team-level performance indicators were chosen to represent multiple dimensions of football performance. Possession rate was retained as a possession-related indicator. Starts, minutes played, and 90s played were retained as tournament-exposure variables. Because these variables were mechanically related to tournament progression, they were excluded from the feature set used in the machine learning models and the Team Strength Score. The 90s played variable was used only to calculate per-90-minute rates. Attacking output indicators included goals and assists. Expected performance indicators included expected goals (xG) and expected assists (xA). Progression indicators included progressive carries and progressive passes. All attacking, expected-performance, and progression indicators were subsequently expressed as per-90-minute rates (Section 2.5) so that tournament exposure did not confound the comparison across teams.

To reduce redundancy, composite or closely overlapping variables, including goals plus assists, non-penalty goals, non-penalty expected goals, and non-penalty expected goals plus expected assists, were excluded from the revised analytical set. Penalty indicators were also excluded from the reduced core set. Yellow and red cards were removed because their performance meaning is context dependent; for example, a tactical foul may sometimes be used to prevent a dangerous transition. Squad size and mean age were retained as descriptive team characteristics but were not treated as performance indicators in the revised analyses.

These indicators were selected because they describe different aspects of team performance. Attacking output indicators reflect scoring and direct goal involvement. Expected performance indicators reflect chance quality and expected attacking contribution. Progression indicators reflect how teams move the ball towards goal and create attacking territory. Possession-related indicators describe the extent to which teams control the ball. The retained indicators correspond to dimensions commonly examined in elite football KPI and match-analysis research, including attacking output, chance quality, possession, and progression (7, 8, 11).

Operational descriptions of the retained variables, based on the FBref source definitions, are provided in Table 1 (17).

**Table 1:** Operational definitions of the retained team-level performance indicators.

**Variable**	**Domain**	**Operational description**	**Analysis form**
Possession (%)	Possession	Team possession percentage reported by FBref.	Percentage
Goals	Attacking output	Goals scored by the team.	Per 90 min
Assists	Attacking output	Assists credited to the team for scored goals.	Per 90 min
xG	Expected performance	Expected goals reported by FBref; a probability-based estimate of shot quality using shot characteristics and preceding events.	Per 90 min
xA	Expected performance	Expected assists reported by FBref; an estimate of the likelihood that a completed pass becomes a goal assist, based on pass characteristics and context.	Per 90 min
PrgC	Progression	Carries that move the ball towards the opponent's goal line at least 10 yards from its furthest point in the last six passes, or any carry into the penalty area; carries ending in the defending 50% of the pitch are excluded.	Per 90 min
PrgP	Progression	Completed passes that move the ball towards the opponent's goal line at least 10 yards from its furthest point in the last six passes, or any completed pass into the penalty area; passes from the defending 40% of the pitch are excluded.	Per 90 min

Definitions reflect the FBref source descriptions used for the exported variables. xG and xA are provider-model estimates, and the definitions are retained to improve reproducibility of the present analysis.

### 2.4 Classification outcome

A binary classification outcome was created to compare the exploratory classification performance of the selected machine learning models. Teams that qualified for the knockout phase of UEFA EURO 2024 were coded as Class 1, whereas teams that failed to qualify from the group phase were coded as Class 0. Class 1 therefore represented teams that progressed to the knockout phase, and Class 0 represented teams that were eliminated in the group phase.

This classification outcome was used only for the supplementary machine learning analysis. The classification analysis was not intended to provide a definitive prediction model for future tournaments. Instead, it was used to examine whether the selected team-level performance indicators could distinguish between teams that progressed beyond the group phase and teams that did not. Because the analysis was based on only 24 team-level observations, the classification results were interpreted cautiously.

### 2.5 Data preprocessing

Data analysis was conducted through data cleaning, selecting features, correlation analysis, exploratory classification, and team performance analysis. First, the dataset was checked for missing values, inconsistent entries, and formatting errors. During this process, a labelling error was identified in the raw exported file: the columns originally labelled ‘Matches Played,’ ‘Starts,’ and ‘Minutes Played' were found to actually contain starts, minutes played, and 90s played, respectively. Column labels were corrected accordingly before any further analysis; a genuine team-level ‘matches played' count was not available in the source export. All selected variables were converted into numerical format when required. Because teams that progressed further in the tournament played more matches and therefore accumulated larger cumulative totals independent of underlying performance quality, all cumulative attacking, expected-performance, and progression indicators were converted to per-90-minute rates (value ÷ 90s played) prior to normalisation. Relevant features were then normalised to preserve comparability across variables measured on different numerical scales.

Min–Max normalisation was applied:

$$X_{norm}=\frac{X_{i}-X_{min}}{X_{max}-X_{min}}$$

where $X_{i}$ represents the observed value of a given performance variable, while $X_{min}$ and $X_{max}$ denote the minimum and maximum values of that variable across all teams.

For the machine learning analysis, Min-Max scaling was fitted within each leave-one-out cross-validation training fold and then applied to the held-out team. For the descriptive Team Strength Score, Min-Max scaling was applied across the full 24-team sample.

### 2.6 Correlation analysis

Pearson correlation coefficients were calculated to examine relationships among the selected team-level performance indicators. The purpose of this analysis was to identify associations among attacking output, expected performance, ball progression, and possession-related variables. Correlation analysis was also used to identify highly related indicators before interpreting the team-level performance results.

The correlation coefficients were interpreted descriptively. The analysis focused on the direction and relative strength of associations rather than on causal inference. This approach was appropriate because the dataset consisted of a small number of team-level observations from a single tournament. Because football KPIs are interdependent and context-sensitive, the coefficients were used to characterise shared variation rather than to identify independent causal determinants (5, 11). Before model fitting, the pairwise correlation matrix was inspected for predictor redundancy. Composite or closely overlapping variables had already been removed from the analytical set, while the remaining correlated indicators were retained because they represented distinct football-specific performance dimensions. Their shared variation was therefore considered when interpreting the model results.

### 2.7 Machine learning models

Four supervised machine learning models [Logistic Regression, Random Forest, Support Vector Machine (SVM), and XGBoost] were applied as supplementary exploratory classifiers. These models were selected because they represent different modelling approaches. Logistic Regression provides a linear and interpretable classification model. Random Forest uses an ensemble of decision trees and can capture non-linear associations. Support Vector Machine identifies a separating boundary between classes. Extreme Gradient Boosting applies sequential decision trees to improve classification performance.

The final feature set comprised possession rate and the per-90-minute rates of goals, assists, xG, xA, progressive carries, and progressive passes. The same feature set was used across all four models. No hyperparameter tuning was performed. Logistic Regression was implemented with max_ite*r* = 1000, the lbfgs solver, L2 regularisation, and C = 1.0. Random Forest used 300 trees, with all other parameters at their default values. SVM used a radial-basis-function kernel with probability = True, C = 1.0, and gamma = 'scale'. XGBoost used eval_metric = ‘logloss', with all other parameters at their default values. A fixed random seed (random_state = 42) was used where applicable to improve reproducibility.

Logistic Regression, Random Forest, and SVM were implemented using scikit-learn version 1.8.0, and XGBoost was implemented using XGBoost version 3.4.1. Model performance was evaluated using accuracy, precision, recall, and F1-score. These metrics were calculated for both classes and were also summarised using macro-averaged and weighted-averaged F1-scores.

### 2.8 Model validation

To evaluate model performance while maximising the training data available in this small sample, leave-one-out cross-validation (LOOCV) was used throughout the machine learning analysis. LOOCV is a special case of k-fold cross-validation in which the number of folds equals the sample size (k = 24). In each iteration, one of the 24 national teams was held out as the test sample while the remaining 23 teams were used for model training. This procedure ensured that every team served as the test sample exactly once.

Min-Max scaling was implemented within a scikit-learn Pipeline together with each classifier. The scaler was therefore fitted only to the 23 training teams in each LOOCV iteration and was subsequently applied to the held-out team. This procedure prevented information from the test observation from entering the preprocessing stage.

Accuracy, precision, recall, and F1-score reported in Table 2 were calculated from the pooled out-of-sample predictions obtained across all 24 LOOCV iterations. Each team therefore contributed exactly one out-of-sample prediction.

**Table 2:** Classification performance of the machine learning models.

Model	Accuracy	Precision (Class 0)	Recall (Class 0)	F1-score (Class 0)	Precision (Class 1)	Recall (Class 1)	F1-score (Class 1)	Macro Avg F1	Weighted Avg F1
Logistic Regression	0.58	0.00	0.00	0.00	0.64	0.88	0.74	0.37	0.49
Random Forest	0.54	0.20	0.12	0.15	0.63	0.75	0.69	0.42	0.51
SVM	0.58	0.00	0.00	0.00	0.64	0.88	0.74	0.37	0.49
XGBoost	0.50	0.00	0.00	0.00	0.60	0.75	0.67	0.33	0.44

Class 1 indicates teams that qualified for the knockout phase of UEFA EURO 2024. Class 0 indicates teams that were eliminated in the group phase.

Because the dataset contained only 24 observations, the machine learning results should be interpreted as exploratory. Although LOOCV maximises the training sample in each iteration, the substantial overlap among training sets may result in unstable performance estimates in a dataset of this size. The small sample size limits the external validity of the models, and their performance should therefore not be interpreted as evidence of generalisable predictive accuracy for future tournaments.

### 2.9 Team strength score

To evaluate the overall competitive performance of each national team, a composite Team Strength Score (TSS) was calculated using normalised performance indicators. Prior to normalisation, cumulative indicators were converted to per-90-minute rates (Section 2.5) so that the TSS reflects performance intensity rather than cumulative tournament exposure. After normalisation, the TSS was calculated as the arithmetic mean of the seven retained performance indicators:

$$TSS=\frac{1}{n}\sum_{i=1}^{n}X_{norm,i}$$

where *n* represents the total number of selected performance indicators.

Attacking output indicators, expected performance indicators, progression indicators, and possession contributed positively to the score. The resulting score provided a multidimensional summary of overall team performance within UEFA EURO 2024. The TSS was used to rank the 24 national teams. It was intended to provide a transparent summary of tournament-level team performance and was not intended to replace detailed tactical analysis or match-specific interpretation. The TSS formulation was developed for the present study. Equal weighting was used to keep the descriptive aggregation transparent rather than to create a validated predictive index. This rationale is consistent with multidimensional KPI research that considers related technical and tactical indicators jointly while emphasising their contextual interpretation (1, 5, 11).

### 2.10 Statistical analysis

Descriptive statistics were calculated for the selected team-level indicators. Pearson correlation coefficients were calculated to examine associations among indicators. Machine learning model performance was evaluated using accuracy, precision, recall, and F1-score. Team Strength Scores were calculated from normalised performance indicators.

All calculations were performed in Python using NumPy, Pandas, Scikit-learn version 1.8.0, and XGBoost version 3.4.1.

## 3 Results

### 3.1 Classification performance of the machine learning models

Table 2 presents the classification performance of the selected machine learning models for the binary outcome of knockout-phase qualification. Tournament-involvement variables, including starts, minutes played, and 90s played, were excluded from the final feature set because of their mechanical association with tournament progression. The accuracy of the four models ranged from 0.50 to 0.58. Logistic Regression and Support Vector Machine (SVM) each achieved an accuracy of 0.58, Random Forest achieved 0.54, and XGBoost achieved 0.50. None of the models exceeded the majority-class baseline accuracy of 0.67, which would be obtained by assigning all teams to Class 1.

The class-specific performance metrics showed that the models generally classified teams that progressed to the knockout phase more accurately than teams eliminated in the group phase. Logistic Regression and SVM did not correctly identify any Class 0 teams. Random Forest showed a Class 0 precision of 0.20 and recall of 0.12, while XGBoost also did not correctly identify any Class 0 teams. Overall, the final models showed limited ability to distinguish teams that progressed to the knockout phase from teams eliminated in the group phase within this 24-team sample.

Figure 1 summarises the classification accuracy of the four models. In the sensitivity analysis, inclusion of tournament-involvement variables increased classification accuracy to 0.96 for Logistic Regression and 1.00, 1.00, and 1.00 for Random Forest, SVM, and XGBoost, respectively. This supported their exclusion from the final feature set because the higher accuracy reflected outcome leakage.

**Figure 1:**
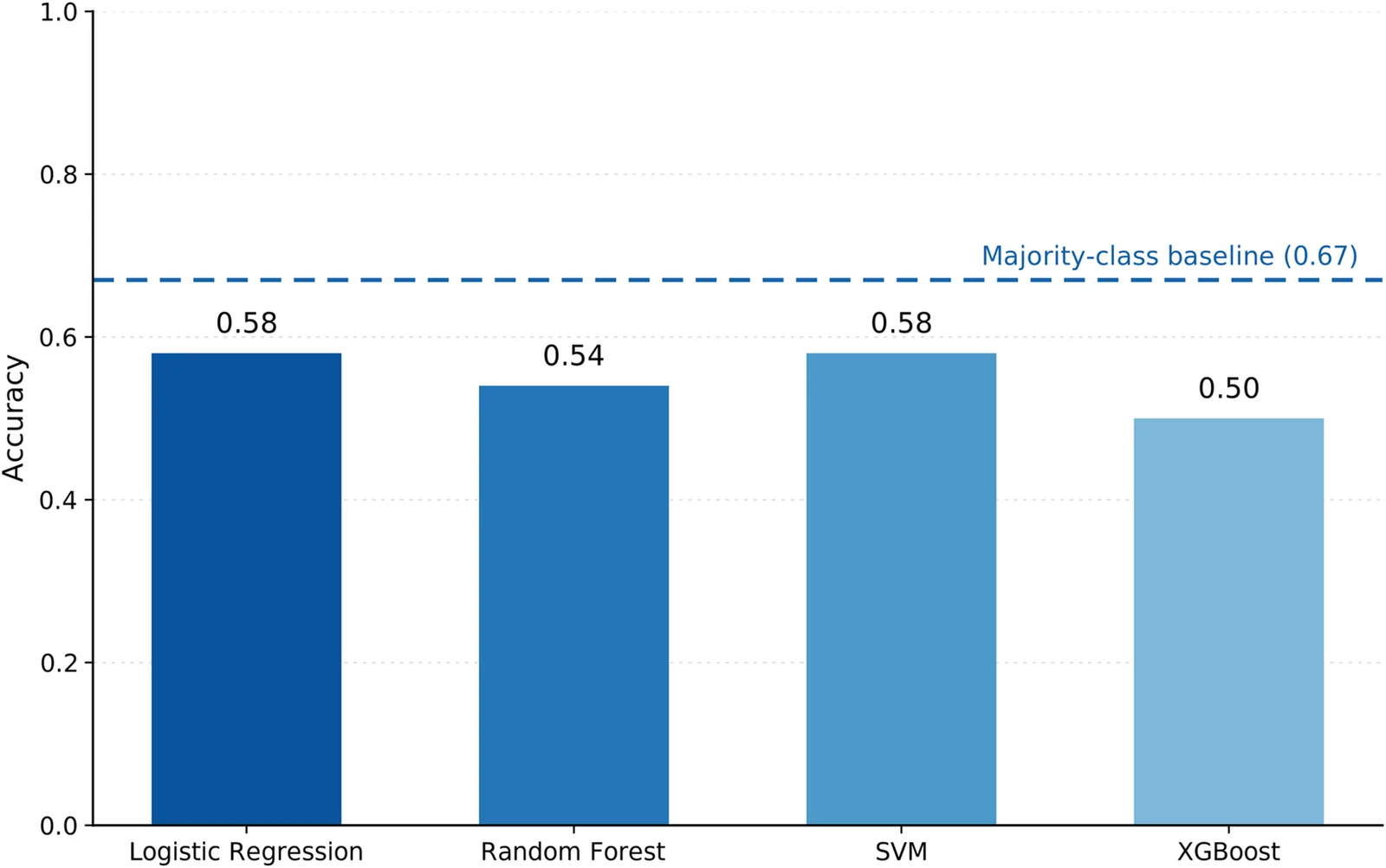
Classification accuracy of the selected machine learning models (leave-one-out CV).

### 3.2 Relationships among team-level performance indicators

Figure 2 shows the correlation heatmap of the selected team-level performance indicators, expressed as per-90-minute rates to remove the confounding influence of tournament exposure. Positive correlations were observed among several attacking output and expected performance indicators. Goals and assists were strongly related (r = 0.91), as were xG and xA (r = 0.90). Progressive carries and progressive passes were also strongly related (r = 0.89).

**Figure 2:**
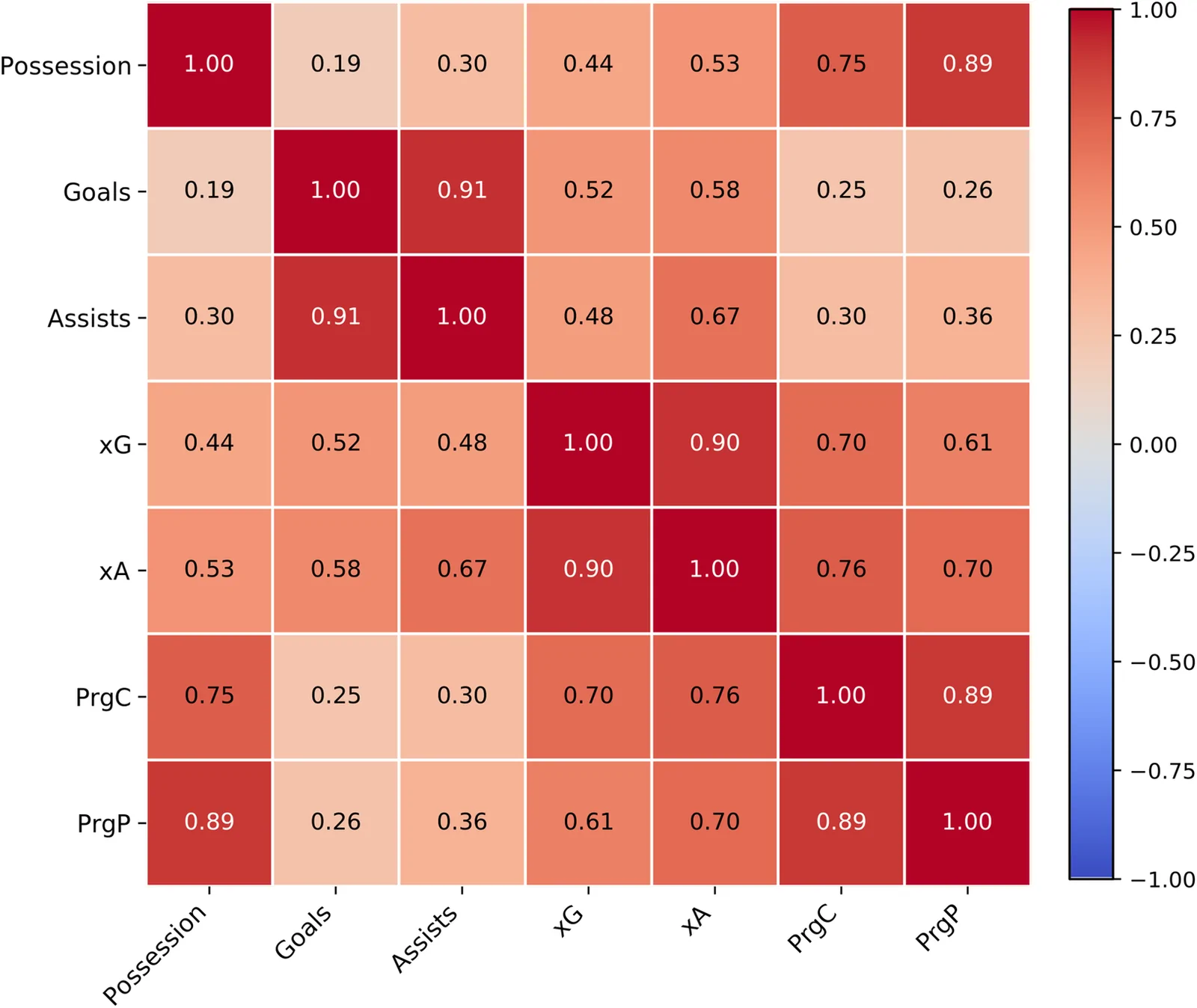
Correlation heatmap of team-level football performance indicators.

Progression indicators also showed positive associations with expected performance and possession. Possession was strongly related to progressive passes (r = 0.89) and progressive carries (r = 0.75), while xA was positively related to progressive carries (r = 0.76) and progressive passes (r = 0.70). By contrast, possession showed lower correlations with goals (r = 0.19) and assists (r = 0.30), and progressive carries showed lower correlations with goals (r = 0.25) and assists (r = 0.30). These findings indicate that attacking output, expected performance, ball progression, and possession were related dimensions of team performance. Some of the strongest coefficients were expected because the underlying actions are operationally linked; for example, progression occurs during possession, and assists are embedded in many goal-scoring sequences.

### 3.3 Clustered correlation network

Figure 3 illustrates the clustered correlation network of the same indicators used in Figure 2, restricted to relationships of |r| ≥ 0.70 for clarity. Goals and assists formed a closely connected pair, as did xG and xA. Progressive carries and progressive passes were strongly connected, and possession was linked to both progression indicators. Because several edges connect conceptually related actions, the network was interpreted as a map of related dimensions rather than as a set of independent predictors. Overall, the network supported the interpretation that attacking output, expected performance, ball progression, and possession were connected dimensions in the UEFA EURO 2024 team-level dataset.

**Figure 3:**
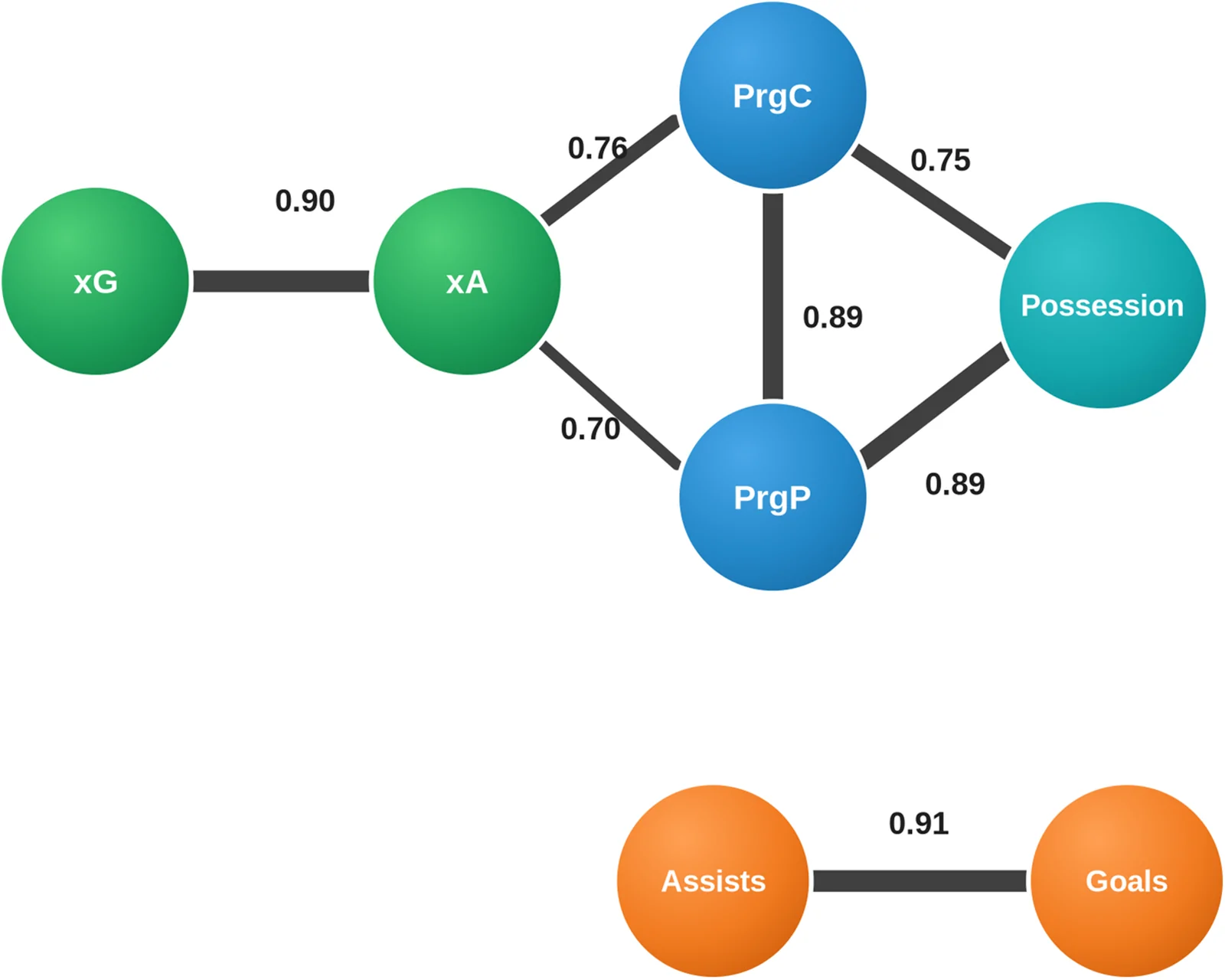
Clustered correlation network of team-level football performance indicators.

### 3.4 Team strength score rankings

Figure 4 presents the TSS rankings derived from the normalised performance indicators. Spain had the highest TSS at 0.88, followed by Germany at 0.86. Austria ranked third with a score of 0.68, followed by Portugal at 0.66 and the Netherlands at 0.62. These rankings should be interpreted as descriptive summaries of the selected indicators rather than as inferential comparisons or probabilities of future tournament success.

**Figure 4:**
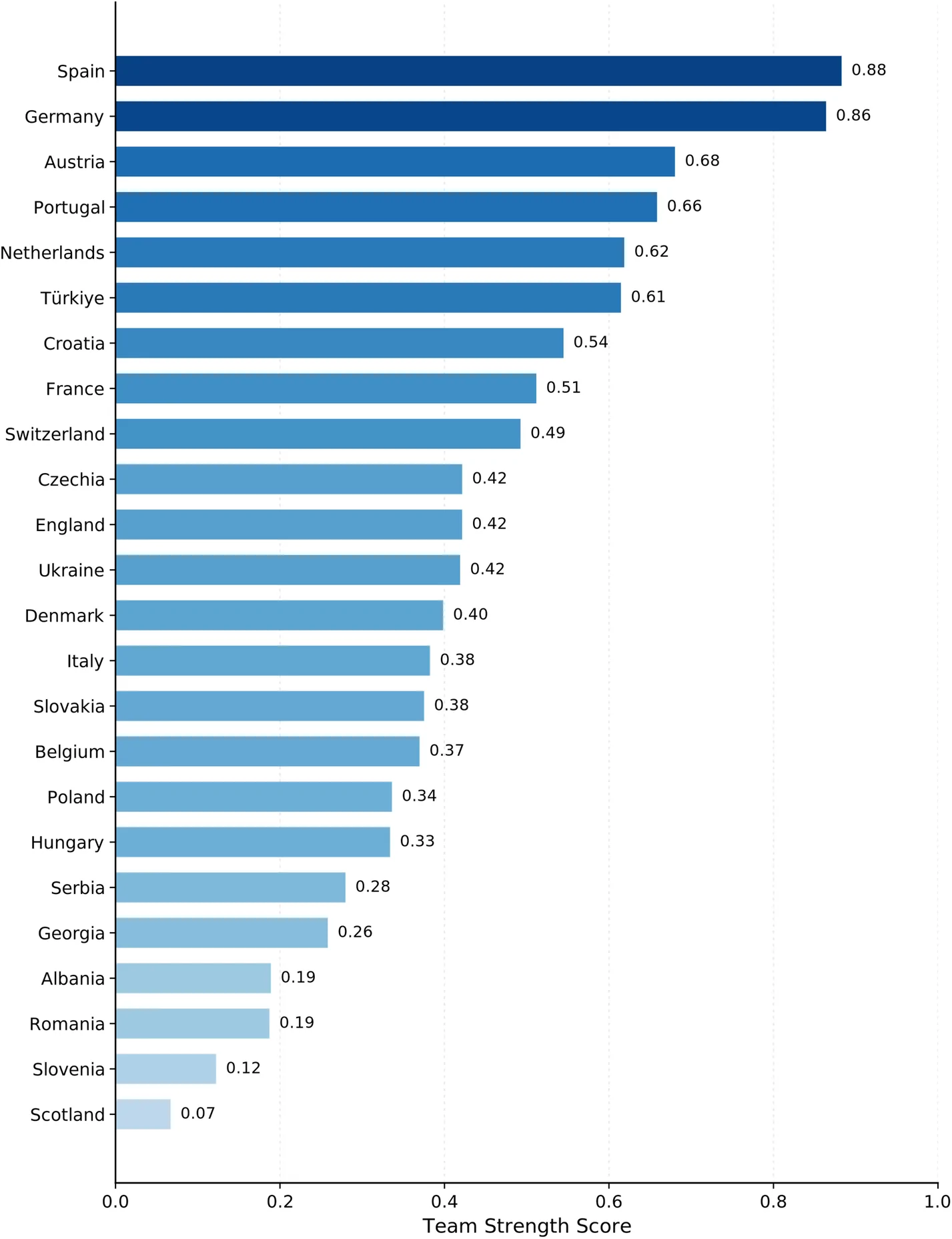
Team strength score rankings.

## 4 Discussion

The present study examined multidimensional team-level indicators from men's UEFA EURO 2024. The main findings were that Logistic Regression, Random Forest, Support Vector Machine, and XGBoost achieved accuracies between 0.50 and 0.58 for the binary outcome of knockout-phase qualification, and none of the models exceeded the 0.67 majority-class baseline. The correlation analysis showed positive associations among attacking output, expected performance, progression, and possession indicators when cumulative indicators were expressed as per-90-minute rates. The TSS identified Spain and Germany as the highest-ranked teams. These findings suggest that multidimensional indicators can support post-tournament comparison, although the results should be interpreted cautiously because the analysis was based on 24 team-level observations from a single tournament.

### 4.1 Interpretation of the classification results

The machine learning models were used as supplementary exploratory classifiers rather than as definitive predictive models. An initial version of this analysis included tournament-involvement variables (starts, minutes played, 90s played) as model features, which produced perfect or near-perfect classification accuracy. Because these variables are mechanically determined by how far a team progressed in the tournament, this represented outcome leakage rather than genuine predictive signal. After excluding tournament-involvement variables and expressing the remaining cumulative indicators as per-90-minute rates, classification accuracy ranged from 0.50 to 0.58 across all four models, and none exceeded the 0.67 majority-class baseline. This result indicates that retained performance indicators alone did not strongly separate teams that progressed to the knockout phase from teams eliminated in the group phase within the analysed sample.

The cautious interpretation of this result is important because the dataset was small and was based on aggregated team-level observations. Classification performance can be sensitive to the definition of the binary outcome, the number of observations, the validation procedure, and whether features that are mechanically linked to the outcome are included or excluded. These issues are particularly important in sport analytics, where machine learning models can produce unstable or over-optimistic results when datasets are small, when outcome leakage is present, or when external validation is limited (12, 16). Therefore, the analysis should be understood as a post-tournament classification analysis based on team-level performance indicators rather than a pre-tournament prediction model. This interpretation is consistent with recent football analytics studies that emphasise both the potential and the limitations of machine learning for outcome modelling in football (4, 15).

### 4.2 Relationships among attacking output, expected performance, progression, and possession indicators

The correlation analysis showed that attacking output indicators were closely related to expected performance and progression indicators. Several of the strongest coefficients were also expected from the structure of the actions themselves. Goals and assists can occur within the same scoring sequence, while progressive passes and carries necessarily occur during possession. Accordingly, the high correlations between goals and assists, possession and progression, and progressive passes and carries should be interpreted partly as intrinsic relationships among related football actions rather than as independent performance effects.

At the same time, the correlation pattern was not uniform across domains. Progressive carries were strongly related to progressive passes (r = 0.89), possession (r = 0.75), xG (r = 0.70), and xA (r = 0.76), but showed substantially lower correlations with goals (r = 0.25) and assists (r = 0.30). Possession showed a similar pattern: it was strongly related to progression but only weakly related to goals (r = 0.19) and assists (r = 0.30). This distinction is practically important because maintaining and advancing possession does not determine whether a sequence produces a high-quality chance or a goal. Expected-performance indicators were more closely related to realised attacking output, with xG correlated with goals (r = 0.52) and xA with assists (r = 0.67), but these relationships were still non-deterministic. xG and xA quantify expected attacking value rather than realised execution, so actual goals and assists may diverge because of finishing, goalkeeper actions, defensive errors, and other match-specific events (7, 8).

This interpretation is consistent with previous work showing that goal-scoring opportunities arise through different tactical routes and that their determinants vary across competitions and phases of play (9–11). It also supports treating possession as a platform for progression rather than a direct proxy for attacking effectiveness, consistent with multidimensional and event-based approaches to football analysis (5, 6). The reduced indicator set addressed mathematical overlap by removing composite variables such as goals plus assists and npxG plus xA, but the remaining correlations still reflect genuine conceptual links among the retained indicators. Therefore, the coefficients should be interpreted descriptively and in conjunction with match context rather than as evidence that each indicator independently determines team success.

### 4.3 Team strength score

The TSS, calculated from the reduced set of normalised performance indicators, provided a transparent summary of the multidimensional team performance. Spain and Germany had the highest TSS, followed by Austria, Portugal, and the Netherlands. These rankings indicate which teams showed relatively strong overall performance across the selected indicators within UEFA EURO 2024, but they should not be interpreted as evidence of a specific tactical style or as inferential comparisons between teams.

The use of a composite score is useful when the aim is to summarise several related performance dimensions into a single interpretable measure. This rationale is consistent with multidimensional approaches in football analytics, in which technical and tactical indicators are considered jointly rather than interpreted in isolation (1, 5). Such an approach can help analysts organise complex performance information and identify dimensions that warrant closer examination. However, a composite score should not replace detailed tactical analysis. Instead, it should be used as an initial benchmarking tool that directs analysts back to the underlying indicators and, subsequently, to match-specific video and contextual analysis.

In practical terms, teams with similar TSS values may reach those scores through different combinations of performance indicators. For example, one team may achieve a relatively high score through stronger possession and progression, whereas another may achieve a similar score through greater attacking output or expected performance. Therefore, the value of the TSS lies not only in the overall ranking but also in examining which indicators contribute to each team's score. This interpretation is consistent with the role of performance analysis as a tool for supporting feedback, preparation, and communication between analysts and coaches (19).

### 4.4 Practical implications

The proposed framework may be useful for performance analysts and coaching staff during post-tournament review. The Team Strength Score can provide an initial overview of how teams compare across multiple performance dimensions. Analysts can then examine the specific indicators that contributed to each team's score. This process may help staff identify whether further examination should focus on attacking output, expected performance, ball progression, or possession.

The framework may also support opponent analysis. For example, high values for progressive passes and xA may warrant closer examination of how a team progresses the ball and creates opportunities through passing sequences. High values for progressive carries may similarly direct attention towards situations in which players advance the ball through individual carrying actions. Conversely, high progression or possession combined with relatively low xG, xA, or realised attacking output may prompt analysts to examine whether possession and territorial progression are being converted into entries into dangerous areas and high-quality scoring opportunities. These statistical patterns do not establish tactical behaviours by themselves, but they can help analysts identify specific questions for subsequent video and match-context analysis.

This staged interpretation is consistent with the broader role of performance analysis in football, which is to support feedback, preparation, and communication between analysts and coaches rather than to reduce performance to isolated numerical indicators (5, 19). Accordingly, the practical value of the present framework lies in combining the initial quantitative summary with contextual information and football-specific interpretation.

The findings may also inform training priorities, although they should not be translated directly into training prescriptions. For example, consistently low progression indicators may encourage staff to examine build-up play and forward ball movement, whereas reasonable possession combined with lower expected attacking contribution may prompt closer analysis of how possession is converted into chance creation. Any training response should then be determined using match video, opponent context, and coaching knowledge. Because the present analysis was based on aggregated tournament-level data, the framework should therefore be used as a starting point for deeper technical-tactical analysis rather than as a stand-alone basis for coaching decisions.

### 4.5 Limitations and future research

This study has several limitations. First, the analysis was based on only 24 team-level observations from a single tournament. This small sample limited the stability and external validity of the machine learning results. Although leave-one-out cross-validation ensured that each team was evaluated once as an out-of-sample observation, none of the models exceeded the majority-class baseline. The classification findings should therefore be interpreted as exploratory and specific to the present dataset. Future studies should include multiple international tournaments and qualification cycles to assess whether the observed patterns are reproducible in independent samples.

Second, the analysis used aggregated team-level indicators. This approach was appropriate for developing a tournament-level analytical framework, but it did not account for match-to-match variation, player-level contributions, tactical adjustments, or contextual factors. Match status, opponent quality, scoreline, substitutions, player availability, venue, and tactical formation may influence team performance indicators. Future studies should incorporate match-level data and relevant contextual variables to determine whether the same indicators remain informative across different competitive situations.

Third, cumulative performance indicators were expressed as per-90-minute rates to reduce the influence of tournament exposure. However, this adjustment does not account for differences in opponent strength, match context, or the stage of the tournament. Future research should examine context-adjusted indicators and opponent-strength-adjusted performance measures.

Fourth, the selected indicators did not capture all relevant dimensions of football performance. Defensive organisation, pressing intensity, transition play, set-piece effectiveness, spatial control, and player interactions were not fully represented. Future research should integrate event data, tracking data, and tactical indicators to develop a more comprehensive analysis of team performance.

Finally, the TSS applied equal weighting to retained indicators. Although the revised set reduced mathematical redundancy, some indicators remained correlated because they describe related performance processes. Future studies should examine alternative weighting or dimension-reduction procedures in larger samples to assess the robustness of the composite rankings.

Despite these limitations, the present study provides a transparent approach for organising multidimensional team-level indicators from an international football tournament. The framework may help analysts summarise team performance and identify questions for subsequent technical-tactical analysis. Further validation with larger and more detailed datasets is needed before the framework can be used for stronger predictive purposes.

## 5 Conclusions

This study examined multidimensional team-level performance indicators from men's UEFA EURO 2024. Exploratory classification performance remained below the majority-class baseline, indicating limited discrimination of knockout-phase qualification from the retained team-level indicators. Correlation analysis showed strong relationships among several conceptually linked actions, including goals and assists, xG and xA, and possession and progression. These associations should not be interpreted deterministically: possession and ball progression describe how teams maintain and advance play, whereas expected-performance indicators describe opportunity quality and may not translate directly into realised goals or assists. The TSS provided a transparent descriptive summary of tournament-level performance, with Spain and Germany showing the highest values. The TSS should be interpreted as a benchmark rather than a tactical profile or calibrated probability of future success. Overall, the proposed framework may support post-tournament benchmarking, opponent analysis, and communication between performance analysts and coaching staff when quantitative indicators are integrated with match-specific context.

## Data Availability

Publicly available datasets were analyzed in this study. This data can be found here: https://fbref.com/en/comps/676/stats/UEFA-Euro-Stats.
